# Fibroblast Growth Factor 23 Is a Strong Predictor of Adverse Events After Left Ventricular Assist Device Implantation

**DOI:** 10.3390/jcdd12080290

**Published:** 2025-07-29

**Authors:** Wissam Yared, Leyla Dogan, Ahsannullah Madad Fassli, Ajay Moza, Andreas Goetzenich, Christian Stoppe, Ahmed F. A. Mohammed, Sandra Kraemer, Lachmandath Tewarie, Ahmad Abugameh, Rachad Zayat

**Affiliations:** 1 Faculty of Medicine, Department of Cardiac Surgery, University Hospital Aachen, RWTH Aachen University, 52074 Aachen, Germany; w.yared@bbtgruppe.de (W.Y.); ahsanfassli@hotmail.de (A.M.F.); amoza@ukaachen.de (A.M.); agoetzenich@abiomed.com (A.G.); amohammed@ukaachen.de (A.F.A.M.); skraemer@ukaachen.de (S.K.); ltewarie@ukaachen.de (L.T.); 2 Department of Radiology, Neuroradiology, Sonography and Nuclear Medicine, Hospital of the Barmherzige Brueder Trier, 54292 Trier, Germany; 3 Medical Affairs, Abiomed GmbH, 52074 Aachen, Germany; 4 Department of Anesthesiology, Intensive Care, Emergency and Pain Medicine, University Hospital Würzburg, 97080 Würzburg, Germany; christian.stoppe@gmail.com; 5 Department of Cardiovascular Surgery, Klinikum Dortmund gGmbH, 44137 Dortmund, Germany; abugameh@online.de; 6 Faculty of Medicine, Witten/Herdecke University, 58455 Witten, Germany

**Keywords:** left ventricular assist device, HeartMate 3, biomarker, fibroblast growth factor 23, predictions, outcomes, adverse events

## Abstract

Heart failure (HF) and left ventricular hypertrophy (LVH) are linked to fibroblast growth factor 23 (FGF23). This study aims to analyze whether FGF23 can predict postoperative outcomes in unselected left ventricular assist device (LVAD) candidates. Methods: We conducted a prospective observational study that included 27 patients (25 HeartMate3 and 2 HeartMateII) with a median follow-up of 30 months. We measured preoperative FGF23 plasma levels and computed the HeartMateII risk score (HMRS), the HeartMate3 risk score (HM3RS) and the EuroSCOREII with respect to postoperative mortality, as well as the Michigan right heart failure risk score (MRHFS), the Euromacs RHF risk score (EURORHFS), the CRITT score with respect to RHF prediction and the kidney failure risk equation (KFRE) with respect to kidney failure. Multivariate logistic regression and receiver operating characteristic (ROC) analyses were performed. Results: In the multivariate logistic regression, preoperative FGF23 level was found to be a predictor of postoperative RHF (OR: 1.37, 95-CI: 0.78–2.38; *p* = 0.031), mortality (OR: 1.10, 95%-CI: 0.90–1.60; *p* = 0.025) and the need for postoperative dialysis (OR: 1.09, 95%-CI: 0.91–1.44; *p* = 0.032). In the ROC analysis, FGF23 as a predictor of post-LVAD RHF had an area under the curve (AUC) of 0.81. Conclusions: FGF23 improves the prediction of clinically significant patient outcomes—such as need for dialysis, RHF and mortality—after HM3 and HMII implantation, as adding FGF23 to established risk scores increased their predictive value.

## 1. Introduction

Many risk scores have been created to predict postoperative mortality and complications in end-stage heart failure (HF) patients who are receiving left ventricular assist devices (LVADs) [1,2].

Considering the limited availability of hospital resources, a simple yet highly predictive evaluation method for postoperative adverse events could offer significant clinical benefits.

In this context, the recent integration of biomarkers with recognized risk models has enabled higher predictive performance regarding postoperative complications [3,4]. It has recently been demonstrated that the preoperative level of fibroblast growth factor 23 (FGF23) is a potent indicator of long-term outcomes, postoperative complications and surgical mortality in patients undergoing elective cardiac surgery [5]. FGF23—a hormone derived from bone—is mostly secreted by osteoblasts and osteocytes but, under pathological conditions or severe stress, it can also be produced by various tissues, including the heart [6,7]. The corresponding FGF receptors are universally expressed and exist in four isoforms (FGF receptors 1–4) [6,8]. FGF23 interacts separately with FGF receptor 4 in the heart [8]. FGF23 plays a pivotal role in the regulation of phosphate and vitamin D metabolism, making it an integral component of the bone–kidney–parathyroid axis [6,9]. Under physiological conditions, the parathyroid glands promote the release of calcium and phosphate from osseous tissue into the circulatory system through parathyroid hormone [6,9]. Consequently, increased phosphate plasma concentrations stimulate FGF23 production, resulting in enhanced renal phosphate excretion and suppression of vitamin D synthesis due to adequate calcium levels in the circulation [6,9]. This axis is widely recognized as significantly imbalanced in patients with chronic renal disease, contributing to detrimental cardiovascular events [6,9,10]. As renal function deteriorates, the ability for phosphate excretion and vitamin D synthesis declines, resulting in elevated parathyroid hormone (due to low calcium) and FGF23 (due to high phosphate). This consequently leads to left ventricular hypertrophy, heart failure and other detrimental cardiovascular occurrences [6,9,10]. In this patient population, routine evaluation of circulatory FGF23 levels is already recommended as a predictive biomarker [11].

In clinical studies, increased FGF23 has been correlated with detrimental cardiovascular remodeling, specifically concerning the onset of left ventricular hypertrophy, alterations in myocyte calcium handling, upregulation of the renin–angiotensin system and facilitation of vascular calcification [12,13,14]. FGF23 has been demonstrated to be a strong predictor of cardiovascular morbidity and mortality in HF patients [15]. Interestingly, a recent study has indicated a correlation between elevated serum levels of FGF23 and pulmonary arterial hypertension (PAH) [16]. To the best of our knowledge, the role of the bone-derived protein hormone FGF23 [17] in patients with LVAD is unknown. Therefore, the present study aimed to analyze the utility of FGF23 as a biomarker for the prediction of postoperative outcomes in a cohort of LVAD patients.

## 2. Materials and Methods

### 2.1. Patients

This was a prospective observational exploratory study, conducted between February 2017 and December 2020. All patients designated for LVAD implantation in the department of cardiac surgery at RWTH University Hospital (Aachen, Germany) in this period were screened. Exclusion criteria were as follows: age under 18 years old, Interagency Registry of Mechanically Assisted Circulatory Support (INTERMACS) level 1, refusal to participate, emergency hospitalization for cardiac surgery, or inability to provide written consent for participation. Ethical approval was obtained from the Research Ethics Committee of RWTH University, Aachen, Germany (EK 151/09). The study was carried out in accordance with the Declaration of Helsinki. All patients who took part in this study gave written permission after being informed about the study. Between February 2017 and December 2020, 66 LVADs were implanted in our department. A total of 27 patients were included in the study after they consented to participate, of which 25 patients received the HeartMate 3 (HM3; Abbott, Chicago, IL, USA) LVAD and 2 patients received the HeartMate II (HMII; Abbott, Chicago, IL, USA) LVAD.

### 2.2. Laboratory Parameters and Measurements

The central laboratory of our hospital was responsible for the processing of blood samples for the purpose of biochemical monitoring. In addition to the routine laboratory measurements, blood samples were taken under standard conditions for regular preoperative laboratory assessments within 24 h prior to surgery. These samples were then centrifuged at a speed of 2800× *g* for 10 min at a temperature of 4 °C.

Supernatants were stored in aliquots at −80 °C. C-terminal FGF23 levels were measured in plasma samples via enzyme-linked immunosorbent assay using a MicroVue Human FGF23 (C-term) kit (lowest cutoff value of 1.5 relative units (RUs)/mL, highest cutoff value of 14,000 RU/mL; Quidel-Immutopics, San Clemente, CA, USA).

### 2.3. Collection of Clinical Data

Information regarding LVAD implantation, demographics and clinical course were obtained from our institutional database. Laboratory data and the incidence of adverse events (e.g., thromboembolic events, hemorrhage, right heart failure (RHF), mortality and acute kidney injury (AKI)) were collected. The INTERMACS 2021 definition [18] was followed to determine the definition of adverse events. The 2024 Kidney Disease Improving Global Outcomes (KDIGO) classification system was used to classify chronic kidney disease [19]. We used the algorithms issued by the American Society of Nephrology and the National Kidney Foundation to determine the estimated glomerular filtration rate (eGFR) [20]. The kidney failure risk equation (KFRE) was calculated [21]. Additionally, we computed existing risk scores for the prediction of outcomes following LVAD implantation: The HeartMate II risk score (HMRS) [22], the HeartMate 3 risk score (HM3RS) [23], the European system for cardiac operative risk evaluation II (EuroSCORE II) [24], the Michigan RHF score (MRHFS) [25], the CRITT RHF score (CRITT) [26], and the European Registry for Patients with Mechanical Circulatory Support (EUROMACS) RHF score (EURORHFS) [27].

### 2.4. Statistical Analysis

Absolute numbers and percentages are employed to represent categorical variables. Categorical variables were analyzed using either the chi-square test or Fisher’s exact test. The normality of the distribution of continuous data was evaluated using the Kolmogorov–Smirnov test. The means ± standard deviations are presented as the unit of measurement for continuous variables that are normally distributed, while medians and (25th, 75th percentile) are used to represent non-normally distributed variables. A logistic regression analysis was performed to determine whether FGF23 predicts postoperative outcomes, including mortality, RHF and AKI requiring postoperative dialysis. We then conducted multivariable logistic regression and incorporated bio-FGF23 and all other established risk scores to determine which parameters continued to be predictors of postoperative outcomes. We conducted receiver operating characteristic (ROC) analysis as a third phase, in order to determine the area under the curve (AUC) for all risk scores and FGF23 individually. Subsequently, we incorporated the FGF23-predicted values into the scores for prediction of survival, renal failure and RHF. The differences between the AUCs were compared using Delong’s test. The significance threshold was established at *p* < 0.05, and all *p*-values were two-tailed. The jamovi project (2020) (jamovi software, version 2.6.3; JAMovi.org; Available online: https://www.jamovi.org/) was used for the descriptive statistics. All other statistical analyses were carried out using the STATA software (StataCorp 2019 LLC, Software: Release 16, College Station, TX, USA). GraphPad Prism (version 10.4.6 for Mac OS X; GraphPad Software, Boston, MA, USA) was employed to visualize selected results.

## 3. Results

### 3.1. Patient Demographics

The patients’ demographics and preoperative clinical data are summarized in Table 1. A total of 27 patients who consented to participate were included in the study. Among the 27 patients included, the median age was 70 (60, 73) years, and 15% were female (Table 1); 11%, 33% and 56% of patients had INTERMACS level of 2, 3 and 4, respectively; 52% of the patients did not have preoperative chronic kidney disease (CKD), 15% of the patients were classified as CKD stage one and 33% were classified as CKD stage three; one patient had preoperative dialysis; 63% of the patients had pulmonary hypertension; and three patients had undergone prior cardiac surgery. Based on echocardiographic measurements, tricuspid annular plane systolic excursion (TAPSE: 13.89 ± 2.24 mm) and tricuspid annular systolic velocity (TASV: 9.44 ± 1.19 cm/s), no patients exhibited severe or moderate right ventricular dysfunction preoperatively (Table 1). The average HM3RS score was 3.75 ± 0.64, the average HMRS was 1.43 ± 0.69, the median EuroSCORE II score was 11.73 (7.51, 17.65)%, the average MRHFS was 0.74 ± 1.35, the average CRITT score was 1.22 ± 0.8, the average EURORHFS was 3.17 ± 2.53 and the average KFRE score was 4.60 ± 5.77% (Table 1).

### 3.2. Postoperative Complications

**Figure 1:**
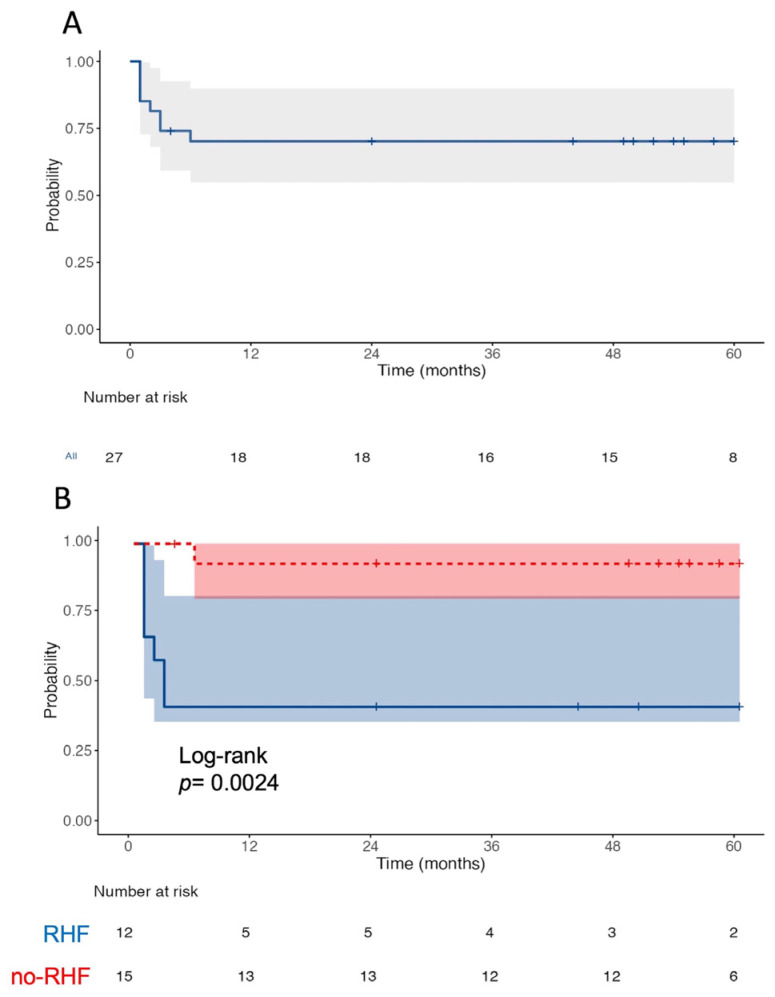
Kaplan–Meier survival curves. (**A**) Kaplan–Meier curve for the whole cohort; (**B**) Kaplan–Meier curves comparing patients with postoperative right heart failure and those without right heart failure.

Postoperative adverse events are summarized in Table 2. The median intensive care unit stay was 6 (4, 22) days. A total of 26% of all patients experienced septic shock, 19% developed postoperative acute kidney injury without requiring dialysis, 26% necessitated temporary dialysis, 44.4% exhibited RHF, and 7.4% required the implementation of a temporary right ventricular assist device (CentriMag, Levitronix LLC, Waltham, MA, USA). Furthermore, 8% of the patients suffered ischemic stroke and 4% had hemorrhagic stroke. The 30-day mortality was 19% and >30-day mortality was 11%. The mean survival time was 42 months (Figure 1A). When comparing those patients who developed post-HMII/3 RHF to those without RHF, patients without post-HMII/3 RHF had a significantly better survival (mean survival: 56 months) than those with RHF (mean survival: 26 months), with log-rank *p* = 0.002 (Figure 1B).

### 3.3. Preoperative Plasma FGF23 Levels Predict Postoperative RHF, Post-HMII/3 Mortality and Post-HMII/3 Dialysis

After multivariable logistic regression with RHF as the dependent variable and consideration of the preoperative FGF23 value, EURORHFS, MRHFS and CRITT scores, the only independent predictor of postoperative RHF after HMII/3 implantation was preoperative FGF23 (OR: 1.37, (95%-CI: 0.78–2.38), *p* = 0.031) (Table 3).

We subsequently conducted multivariable logistic regression with post-HMII/3 mortality as the dependent variable, incorporating the preoperative FGF23 plasma level and HM3RS, HMRS and EuroSCORE II scores. We found that preoperative FGF23 level was the sole predictor of post-HMII/3 mortality (OR: 1.10, (95%-CI: 0.90–1.60), *p* = 0.025) (Table 3).

We also examined whether preoperative FGF23 levels can predict post-HMII/3 dialysis. Unlike KFRE, preoperative FGF23 plasma levels remained the only significant predictor of post-HMII/3 dialysis (OR: 1.09, (95%-CI: 0.90–1.40), *p* = 0.032) (Table 3).

### 3.4. Receiver Operating Characteristic Analysis

**Figure 2:**
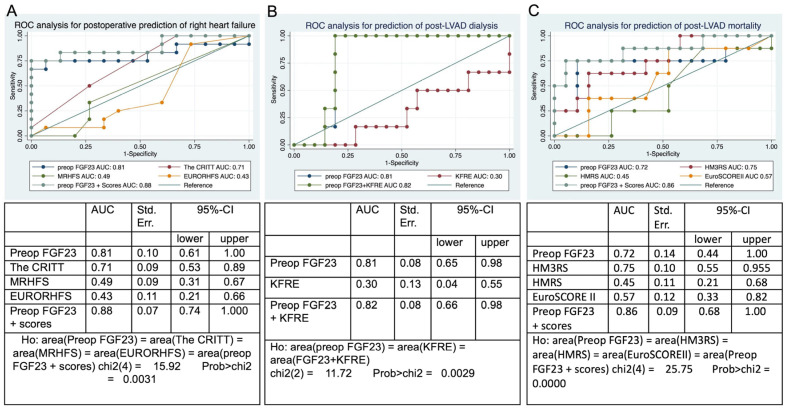
ROC analysis of FGF23 and other risk scores for the prediction of postoperative right heart failure, dialysis and mortality. (**A**) Postoperative right heart failure, (**B**) dialysis, and (**C**) mortality. AUC: area under the curve; CI: confidence interval; EURORHFS: EUROMACS right heart failure risk score; EuroSCORE II: European system for cardiac operative risk evaluation II; FGF23: fibroblast growth factor 23; HMRS: the HeartMate II risk score; HM3RS: the HeartMate 3 risk score; MRHFS: the Michigan RHF score; KFRE: kidney failure risk equation; preop: preoperative; St. Err.: standard error.

In the subsequent phase, we conducted ROC analysis to determine the AUC of preoperative FGF23 in comparison to those of the considered risk scores (CRITT, MRHFS, EURORHFS) for the prediction of postoperative RHF. Subsequently, we integrated FGF23 with the recognized scores to assess its ability to improve their predictive performance. As a predictor of post-HMII/3 RHF, FGF23 had an AUC of 0.81 with sensitivity of 75% and specificity of 93%, the CRITT score had an AUC of 0.71, the MRHFS had an AUC of 0.49 and the EURORHFS had an AUC of 0.43. Upon including the preoperative FGF23 into the existing risk scores, the AUC reached 0.88, exhibiting a sensitivity of 83.3% and a specificity of 86.7% (Figure 2A). Furthermore, Delong’s test indicated a substantial difference in the ROC curves (*p* = 0.003).

We conducted further ROC analysis to predict postoperative dialysis, yielding an AUC of 0.30 for KFRE and an AUC of 0.81 for preoperative FGF23 levels with sensitivity of 100% and specificity of 81%. Upon integrating preoperative FGF23 with the KFRE, the AUC reached 0.82, exhibiting a sensitivity of 100% and a specificity of 81% (Figure 2B). Additionally, a substantial difference between the ROC curves was revealed according to Delong’s test (*p* = 0.002).

The same methodology was employed to compute the AUC for preoperative FGF23, HMRS, HM3RS and the EuroSCORE II, with post-HMII/3 mortality as the dependent variable. The AUC values for EuroSCORE II, HMRS, HM3RS and preoperative FGF23 were 0.57, 0.45, 0.75 and 0.72, respectively, with a sensitivity of 75% and specificity of 89%. The AUC of preoperative FGF23 + the risk scores reached 0.86, with a sensitivity of 75% and specificity of 95%. Delong’s test revealed a significant difference in the ROC curves (*p* < 0.001) (Figure 2C).

## 4. Discussion

This investigation is, to our knowledge, the first prospective observational study in the literature examining the value of FGF23 as a predictive tool for predicting mortality, RHF and the need of dialysis after HMII/3 implantation. The data presented in our study demonstrate that FGF23 enhances the risk stratification ability for RHF, AKI and mortality in an unselected patient population undergoing HMII/3 implantation. As such, it promotes individualized patient care in this highly vulnerable patient population. FGF23 exhibited better discriminatory power when compared to well-established risk scores, such as the CRITT, MRHFS and EURORHFS, for the prediction of post-HMII/3 RHF. FGF23 also showed better predictive ability for post-HMII/3 mortality than the well-known risk scores HM3RS, HMRS and EuroSCORE II. Additionally, preoperative FGF23 levels predicted post-HMII/3 AKI with the need for dialysis better than the KFRE.

As a glycoprotein, FGF23 is part of the fibroblast growth factor family, members of which perform diverse functions in the human body—including the regulation of metabolic processes and organogenesis during embryonic development—mediated by binding to specific FGF receptors with intracellular tyrosine kinase activity [6,28].

Multiple studies have indicated that increased FGF23 levels are correlated with cardiovascular disorders (such as heart failure, atrial fibrillation, myocardial infarction and stroke) and an increased risk of both cardiovascular and non-cardiovascular mortality [5,6,12,13,14].

Our findings regarding the predictive value of preoperative FGF23 levels regarding post-HMII/3 mortality are similar to those reported by Speer et al. [5], who found—in a prospective cohort study involving 859 patients undergoing cardiac surgery—that preoperatively measured FGF23 levels possess equivalent prognostic significance to the established EuroSCOREII in predicting postoperative complications (e.g., AKI and nonocclusive mesenteric ischemia) as well as overall outcomes. In our study, preoperative FGF23 was the only independent predictor of post-HMII/3 mortality when included in a multivariate regression model together with HM3RS, HMRS and EuroSCOREII. Furthermore, in the ROC analysis, preoperative FGF23 had the highest AUC of 0.72 with sensitivity of 75% and specificity of 89%. Most importantly, when we integrated preoperative FGF23 with HM3RS, HMRS and EuroSCOREII, the AUC was increased, reaching a value of 0.86 with sensitivity of 75% and specificity of 95%, thus indicating improved predictive performance.

Our findings are also consistent with those of Hofer et al. [6], who found—in a prospective study including 451 patients undergoing elective coronary artery bypass graft and/or cardiac valve surgery—that preoperative FGF23 serves as an independent predictor of cardiovascular mortality, HF hospitalization and postoperative atrial fibrillation in patients undergoing cardiac surgery.

Similar to our findings regarding the predictive ability of preoperative FGF23 regarding the post-HMII/3 need for dialysis, Speer et al. [5] have also shown that FGF23 is a recognized biomarker for the advancement of CKD; notably, the aforementioned authors showed that FGF23 is strong predictor of AKI after elective cardiac surgery [5].

The strongest evidence comes from patients undergoing dialysis, where FGF23 has been shown to correlate with a high mortality rate [29].

Interactions between the kidney, heart and bone occur in the presence of AKI.

Numerous recently published studies have illustrated the impact of acute kidney injury (AKI) on the susceptibility of cardiomyocytes to the induction of arrhythmias and a heart failure phenotype, which is correlated with elevated levels of FGF23 and reduced Klotho levels. Klotho—an anti-aging protein—is primarily synthesized in the kidneys and exists in both membrane-bound and soluble forms. It is essential for the regulation of multiple physiological functions, including the metabolism of minerals and vitamin D [29,30]. In patients with HF and lower ejection fraction, FGF23 has been shown to possess independent diagnostic potential for the prediction of worse outcomes [31]. Considering that several studies have identified a correlation between FGF23 and decreased ejection fraction (EF), increased plasma levels of FGF23 are believed to be directly associated with systolic dysfunction. Furthermore, a correlation has been observed between patients exhibiting a more severe New York Heart Association score and elevated FGF23 levels [31,32].

While several studies have investigated the relationships between FGF23 expression and left ventricular remodeling characteristics [13,14,33], there remains a lack of information regarding the correlation between FGF23 and right ventricular remodeling. In a limited group of pulmonary hypertension (PHT) patients (n = 48), FGF23 levels exhibited correlations with mean pulmonary artery pressure (mPAP), cardiac index (CI), pulmonary vascular resistance (PVR), NT-proBNP and the REVEAL risk score [34].

In our study, we found that the preoperative FGF23 level is a strong predictor of post-HMII/3 RHF. Widmann and colleagues [15], in their observational research, analyzed plasma FGF23 levels in individuals with PHT (n = 627), dilated cardiomyopathy (n = 59) or left ventricular hypertrophy with severe aortic stenosis (n = 35), while participants without left ventricular or right ventricular dysfunction served as controls (n = 36). Widmann et al. [15] found that FGF23 plasma levels were higher in PHT patients than in healthy controls. Furthermore, they found that patients with high FGF23 plasma levels had higher PVR, mPAP and RASP and lower CI than those with low FGF23 plasma values, which could partially explain the abovementioned observation made in the present study. Additionally, they demonstrated that higher FGF23 levels were associated with higher right ventricular end-diastolic diameter, lower TAPSE and TAPSE/PASP. ROC analysis revealed FGF23 as a good predictor of RV maladaptation.

### Strength and Limitations of the Study

The advantages of our study comprise (a) the standardized assessment of adverse events conducted by experienced intensive care physicians and cardiothoracic surgeons, (b) the external evaluation of biomarkers by individuals unaware of the patient’s clinical course, and (c) the focus on preoperative biomarker values.

We recognize many limitations of our study. The limited sample size constrains the generalizability of the results, allowing our findings to exclusively inform hypotheses. The sample size prevents any conclusive results for high-risk patients due to the complexities of surgery and comorbidities. As such, the diagnostic and prognostic effectiveness of FGF23 should be confirmed through prospective trials including a substantial cohort of HMII/3 patients. As we only analyzed patients receiving the HMII or HM3, our results cannot be extrapolated to all LVAD patients. Ultimately, our results cannot be generalized to chronic HF patients, nor are they relevant to those with moderate decompensated HF, as our study exclusively involved patients with end-stage HF necessitating HMII/3 placement. The confidence interval denotes the degree of uncertainty surrounding the measure of effect—specifically, the precision of the effect estimate—which, in this instance, is represented as an odds ratio (OR). Confidence intervals were employed due to the recruitment of a limited sample from the general population, allowing us to infer that the genuine population effect resides between the upper and lower confidence limits. A 95% confidence interval for an odds ratio that includes 1—which was the case in our study—indicates that the observed data are compatible with a positive association (odds ratio > 1), a negative association (odds ratio < 1), or no association (odds ratio = 1). One possible explanation is the very small group size considered in our study. Therefore, our results must be interpreted with caution.

## 5. Conclusions

In the current study, preoperative FGF23 levels were found to serve as an effective biomarker for predicting post-HMII/3 right heart failure, postoperative acute kidney injury requiring dialysis and death. Furthermore, integrating the preoperative FGF23 level with relevant established risk scores yielded an increased AUC and enhanced predictive capability, indicating improved predictive performance.

The evaluation of FGF23 in patients with HMII/3 may provide essential insights for therapeutic decision making, thus helping to reduce mortality, acute kidney injury and right heart failure in such patients.

## Figures and Tables

**Table 1:** Patient demographic, risk score, preoperative laboratory, echocardiography, right heart catheterization, and perioperative data.

Characteristic	N = 27
Age years	70 (60, 73)
Female n (%)	4 (15%)
BMI kg/m^2^	29.7 (25.2, 33.6)
BSA m^2^	2.09 (1.99, 2.34)
IDDM n (%)	7 (26%)
PAD n (%)	3 (11%)
CVD n (%)	5 (19%)
aHT n (%)	26 (96%)
Nicotine n (%)	15 (56%)
EF (%)	17.9 ± 2.9%
Heartmate 3 n (%)	25 (92.6%)
Heartmate II n (%)	2 (7.4%)
TIA n (%)	2 (7.4%)
HLP	10 (37%)
CKD stages	
0 n (%)	14 (52%)
1 n (%)	4 (14.8%)
3 n (%)	9 (33%)
Preoperative dialysis n (%)	1 (3.7%)
COPD	
0 n (%)	24 (89%)
2 n (%)	1 (3.7%)
3 n (%)	2 (7.4%)
PHT n (%)	17 (63%)
Prior cardiac surgery n (%)	3 (11%)
**Risk scores**	
INTERMCS 2 n (%)	3 (11%)
INTERMCS 3 n (%)	9 (33%)
INTERMCS 4 n (%)	15 (56%)
CRITT	1.22 ± 0.8
MRHFS	0.74 ± 1.35
EURORHFS	3.17 ± 2.53
HM3RS	3.75 ± 0.64
KFRE (%)	4.60 ± 5.77%
HMRS	1.43 ± 0.69
EuroSCOREII (%)	11.73 (7.51, 17.65)
**Echocardiography**	
LVEDD cm	6.24 ± 0.55
TAPSE mm	13.89 ± 2.24
TASV cm/s	9.44 ± 1.19
**Preop laboratory**	
Creatinine mg/dL	1.43 (1.08, 1.72)
INR	1.02 (0.97, 1.07)
Hemoglobin mg/dL	11.17 ± 2.38
Thrombocyte/nL	227 (194, 321)
Albumin g/dL	3.90 (3.60, 4.20)
AST U/L	23 (21, 33)
Hematocrit (%)	37.6 (34.4, 41.5)
BUN mg/dL	56 (41, 62)
Bilirubin mg/dL	0.44 (0.39, 0.60)
**FGF23 plasma levels**	
Preoperative FGF23 RU/mL	243 (105, 1211)
ICU FGF23 RU/mL	1474 (391, 6790)
FGF23 24 h RU/mL	1791 (800, 11,900)
**Preoperative right heart catheterization**	
CO L/m	3.79 ± 1.10
CVP mmHg	13.96 ± 5.72
mPAP mmHg	31.78 ± 15.82
SPAP mHg	45.48 ± 15.07
PCWP mmHg	23.74 ± 10.63
**Perioperative data**	
OP-Zeit minutes	239.16 ± 72.66
Bypass-Zeit minutes	110.96 ± 45.09
Cross-Clamp Zeit minutes	9.12 ± 9.88

AHT: arterial hypertension; AST: aspartate aminotransferase; BMI: body mass index, Kg/m^2^; BSA: body surface area, m^2^; BUN: blood urea nitrogen; CPB: cardiopulmonary bypass; CKD: chronic kidney disease; COPD: chronic obstructive pulmonary disease; CVD: cerebrovascular disease; CVP: central venous pressure; DCM: dilative cardiomyopathy; EF: ejection fraction; EURORHFS: EUROMACS right heart failure score; EuroSCORE II: European system for cardiac operative risk evaluation II; HMRS: the HeartMate II risk score; HM3RS: the HeartMate 3 risk score; IDDM: insulin-induced diabetes mellitus; ICM: ischemic cardiomyopathy; HLP: hyperlipoproteinemia; INR: international normalized ratio; INTRMACS: Interagency Registry for Mechanically Assisted Circulatory Support; mPAP: mean pulmonary artery pressure; MRHFS: the Michigan RHF score; PAD: peripheral arterial disease; PHT: pulmonary hypertension; TASV: tricuspid annular systolic velocity; TAPSE: tricuspid annular plane systolic excursion; TIA: transitory ischemic attack; postop: postoperative; SPAP: systolic pulmonary pressure; PCWP: postcapillary wedge pressure.

**Table 2:** Postoperative complications and laboratory data.

ICU stay days	6 (4, 22)
ICU readmission n (%)	6 (22%)
Hospital LOS days	21 (13, 55)
mechanical ventilation duration h	168 (16, 912)
30-day mortality n (%)	5 (19%)
>30-day mortality n (%)	3 (11%)
Postoperative pneumonia n (%)	14 (52%)
Post-sepsis n (%)	7 (26%)
Delirium n (%)	7 (26%)
AKI with dialysis n (%)	7 (26%)
AKI without dialysis n (%)	5 (19%)
Re-thoracotomy n (%)	11 (41%)
RHF n (%)	12 (44.4%)
RVAD implantation n (%)	2 (7.4%)
iNO inhalation h	14 (0, 53)
Device thrombus n (%)	0
Ischemic stroke n (%)	2 (8.0%)
Hemorrhagic stroke	1 (4.0%)
**Laboratory parameters before hospital discharge**	
fHb mg/dL	40 (27, 60)
Hb g/dL	10.20 (8.90, 10.90)
Platelet count/nL	274 (170, 330)
INR	2.50 (2.10, 2.90)
LDH U/L	294 (254, 349)
AST U/L	48 (20, 52)
ALT U/L	40 (15, 60)
Bilirubin mg/dL	0.34 (0.33, 0.44)
Creatinine mg/dL	1.19 (0.88, 1.82)
BUN mg/dL	56 (50, 66)
Albumin g/dL	2.80 (2.20, 2.90)

AKI: acute kidney injury; ALT: alanine aminotransferase; AST: aspartate aminotransferase; BUN: blood urea nitrogen; fHb: free plasma hemoglobin; Hb: hemoglobin; ICU: intensive care unit; iNO: inhaled nitroxide; INR: international normalized ratio; LDH: lactate dehydrogenase; LOS: length of stay; RVAD: right ventricular assist device.

**Table 3:** Multivariable logistic regression results with FGF23 and other risk scores as predictors of right heart failure, mortality and post-HMII/3 dialysis.

**Postoperative Right Heart Failure as Dependent Variable**					95% Confidence Interval	
Predictor	Estimate	SE	*p*	Odds Ratio	Lower	Upper
Intercept	2.57	1.53	0.092	13.11	0.66	261.69
Preop FGF23	0.31	0.28	0.031	1.37	0.78	2.38
The CRITT	−0.89	1.09	0.412	0.41	0.05	3.45
EURORHFS	−0.00	0.00	0.270	1.00	0.99	1.00
MRHFS	−0.08	0.54	0.882	0.92	0.32	2.67
**Post-HMII/3 mortality as dependent variable**					95% Confidence Interval	
Predictor	Estimate	SE	*p*	Odds ratio	Lower	Upper
Intercept	−10.21	5.23	0.051	0.00	0.00	1.04
Preop FGF23	0.00	0.00	0.025	1.10	0.90	1.60
HM3RS	1.82	1.07	0.088	6.20	0.76	50.49
HMRS	0.65	1.14	0.568	1.92	0.20	18.06
EuroSCOREII	−0.01	0.04	0.735	0.99	0.92	1.06
**Post-HMII/3 dialysis as dependent variable**					95% Confidence Interval	
Predictor	Estimate	SE	*p*	Odds ratio	Lower	Upper
Intercept	−2.48	1.16	0.032	0.08	0.01	0.80
Preop FGF23	0.00	0.00	0.032	1.09	0.91	1.44
KFRE	−0.11	0.16	0.487	0.90	0.66	1.22

EURORHFS: The European Registry for Patients with Mechanical Circulatory Support right heart failure risk score; EuroSCORE II: European system for cardiac operative risk evaluation II; FGF23: fibroblast growth factor 23; HMRS: the HeartMate II risk score; HM3RS: the HeartMate 3 risk score; MRHFS: the Michigan RHF score; KFRE: kidney failure risk equation; LVAD: left ventricular assist device; preop: preoperative; SE: standard error.

## Data Availability

The data supporting this publication are available from the corresponding author upon reasonable request.

## Abbreviations

The following abbreviations are used in this manuscript:

95%-CI	95% confidence interval
AHT	Arterial hypertension
AST	Aspartate aminotransferase
AKI	Acute kidney injury
AUC	Area under the curve
BMI	Body mass index Kg/m^2^
BSA	Body surface area m^2^
BUN	Blood urea nitrogen
Bio-ADM	Bioactive adrenomedullin
CPB	Cardiopulmonary bypass
CKD	Chronic kidney disease
COPD	Chronic obstructive pulmonary disease
CRITT score	CVP greater than 15 mm Hg (C); severe RV dysfunction (R); preoperative mechanical ventilation/intubation (I); severe tricuspid regurgitation (T); and tachycardia (T)
CVD	Cerebrovascular disease
CVP	Central venous pressure
DCM	Dilative cardiomyopathy
EDTA	Ethylenediaminetetraacetic acid blood sample
EuroSCORE II	European system for cardiac operative risk evaluation II
EURORHFS	The European Registry for Patients with Mechanical Circulatory Support right heart failure score
FGF-23	Fibroblast growth factor 23
IDDM	Insulin-dependent diabetes mellitus
HLP	Hyperlipoproteinemia
HMII	HeartMate II
HM3	HeartMate 3
HMRS	The HeartMate II risk score
HM3RS	The HeartMate 3 risk score
HF	Heart failure
ICU	Intensive care unit
iNO	Inhaled nitroxide
INTERMACS	the Interagency Registry for Mechanically Assisted Circulatory Support
INR	International normalized ratio
ICM	Ischemic cardiomyopathy
KFR	kidney failure risk score
LVADs	Left ventricular assist devices
LOS	Length of stay
MRHFS	Michigan-right-heart-failure risk score
mPAP	Mean pulmonary artery pressure
OR	Odds ratio
PAD	Peripheral arterial disease
Penkid	Proenkephalin A
PHT	Pulmonary hypertension
RAP/PCWP	Right atrium pressure/postcapillary wedge pressure ratio
ROC	Receiver operating characteristic analysis
RHF	Right heart failure
RVAD	Right ventricular assist device
RVFAC	RV fractional area change
sPAP	Systolic pulmonary pressure
TASV	Tricuspid annular systolic velocity
TAPSE	Tricuspid annular plane systolic excursion
TIA	Transitory ischemic attack
